# Baseline serum ferritin predicts myocardial iron uptake following intravenous iron therapy – a hypothesis‐generating study

**DOI:** 10.1002/ejhf.3730

**Published:** 2025-06-16

**Authors:** Julio Nunez, Anna Mollar, Mayra Vera‐Aviles, Syeeda Kabir, Akshay Shah, Paolo Polzella, Michael Desborough, Ingrid Cardells, Gema Miñana, Irene del Canto, Vanessa Ferreira, Stefan Piechnik, Alicia Maceira, Samira Lakhal‐Littleton

**Affiliations:** ^1^ Hospital Clínico de Valencia, Fundación INCLIVA Valencia Spain; ^2^ Universidad de Valencia Valencia Spain; ^3^ CIBER Cardiovascular Madrid Spain; ^4^ Department of Physiology, Anatomy and Genetics University of Oxford Oxford UK; ^5^ Nuffield Department of Clinical Neurosciences University of Oxford Oxford UK; ^6^ Department of Clinical Haematology Oxford University Hospitals NHS Foundation Trust Oxford UK; ^7^ Hospital de Manises Valencia Spain; ^8^ Oxford Centre for Clinical Magnetic Resonance Research (OCMR) University of Oxford Oxford UK; ^9^ ASCIRES Valencia Spain

## Introduction

Myocardial iron uptake has been proposed as one of the mechanisms underlying the benefits of intravenous (IV) iron therapy in patients with iron deficiency (ID) and heart failure (HF). However, it is unknown whether myocardial iron uptake relates to baseline iron markers. Myocardial iron uptake can be determined non‐invasively as decreased T1 or T2* in cardiac magnetic resonance (CMR). Using this approach, the Myocardial‐IRON trial previously showed that treatment with IV ferric carboxymaltose (FCM) in patients with HF significantly decreased myocardial T1 and T2* at 7 days, together with improvement in global longitudinal strain at 30 days.^1^, ^2^ More recently, a pharmacokinetic trial (STUDY) in patients with ID without HF demonstrated that FCM decreased myocardial T1 and this decrease was sustained at 42 days.^3^ STUDY also reported a marked decrease in spleen T1 at 14 days, which was partially reversed at 42 days, confirming the known role for spleen macrophages in the uptake and subsequent redistribution of iron from iron‐carbohydrate complexes such as FCM.

The aim of this study was to examine the relationship between baseline iron markers and myocardial iron uptake after IV iron therapy with FCM, and the role of the spleen in this context. Understanding this relationship could help inform the ongoing debate over what markers best predict favourable response to IV iron therapy.

## Methods

### Study population and procedures

These are post‐hoc analyses of the Myocardial‐IRON and STUDY trials.^1^, ^2^, ^3^ Details of study populations were reported previously.^1^, ^2^, ^3^

Myocardial‐IRON was a multicentre, randomized, double‐blind, placebo‐controlled trial to assess the effect of IV FCM versus placebo on myocardial iron repletion at 7 and 30 days in patients with HF and ID (*n* = 53). Detailed information about the study was published elsewhere.^1^, ^2^ This study is registered at ClinicalTrials.gov (NCT03398681).

STUDY was an investigator‐initiated, single‐centre, prospective, observational study. It included 12 patients without HF receiving FCM as standard of care for the correction of ID. Detailed information and main results were published elsewhere.^3^ The study was registered prospectively on the ISRCTN registry (ISRCTN15770553) and ClinicalTrials.gov (NCT05609318).

### Statistical analysis

Association of baseline ferritin and transferrin saturation (TSAT) with baseline T1, T2*, and three‐dimensional global longitudinal strain (3D‐GLS) were assessed using Spearman correlation coefficients. In the Myocardial‐IRON trial, relationships between baseline ferritin and TSAT and changes in T1 (∆T1), changes in T2* (∆T2*) and changes in global longitudinal strain (Δ3D‐GLS) were assessed using linear mixed‐effect models, adjusted for baseline value of the endpoints (ANCOVA design), age, sex, and participant sites. In the STUDY cohort, within‐group unadjusted comparisons using linear regression was used to examine the association of baseline ferritin and TSAT with ∆T1 and ∆T2* at 42 days. Estimates were presented as least square means with 95% confidence intervals. A two‐sided *p* < 0.05 was considered significant, and no adjustments were made for multiple comparisons. All analyses were performed using STATA 18.0 (Stata Statistical Software, College Station, TX, USA).

## Results

In Myocardial‐IRON, the median age of the sample was 73 years, with 75.5% men, 94.3% patients in New York Heart Association class II and 5 (9.4%) on stable treatment with empagliflozin 10 mg/day at baseline. Median N‐terminal pro‐B‐type natriuretic peptide was 1690 pg/ml, and all patients fulfilled the European Society of Cardiology ID criteria. The median (Q1 to Q3) ferritin and TSAT were 63.0 μg/L (33.1–114.3) and 15.7% (11.0–19.2), respectively. Baseline mean 3D‐GLS was −5.6 ± 4.1%. There were no significant differences between treatment groups at baseline. In STUDY, of the 12 participants enrolled, 11 completed a scan at 42 days post‐FCM treatment. In these, the median age was 47 years, and 91% were women. Median ferritin at referral, and baseline TSAT were 7.4 μg/L (5.3–18.1) and 10.0% (7.8–25.7), respectively.

In terms of associations between variables at baseline, in Myocardial‐IRON, baseline serum iron markers were not correlated with either baseline myocardial T1 (*r* = 0.02, *p* = 0.903 for ferritin and *r* = −0.17, *p* = 0.216 for TSAT) or baseline myocardial T2* (*r* = −0.08, *p* = 0.552 for ferritin and *r* = −0.19, *p* = 0.153 for TSAT). In STUDY, baseline ferritin correlated with baseline myocardial T1 (*r* = −0.708, *p* = 0.014) and with baseline myocardial T2* (*r* = −0.674, *p* = 0.026). Baseline TSAT in STUDY did not correlate with either baseline myocardial T1 (*r* = −0.301, *p* = 0.370) or baseline myocardial T2* (*r* = −0.218, *p* = 0.519).

**Figure 1:**
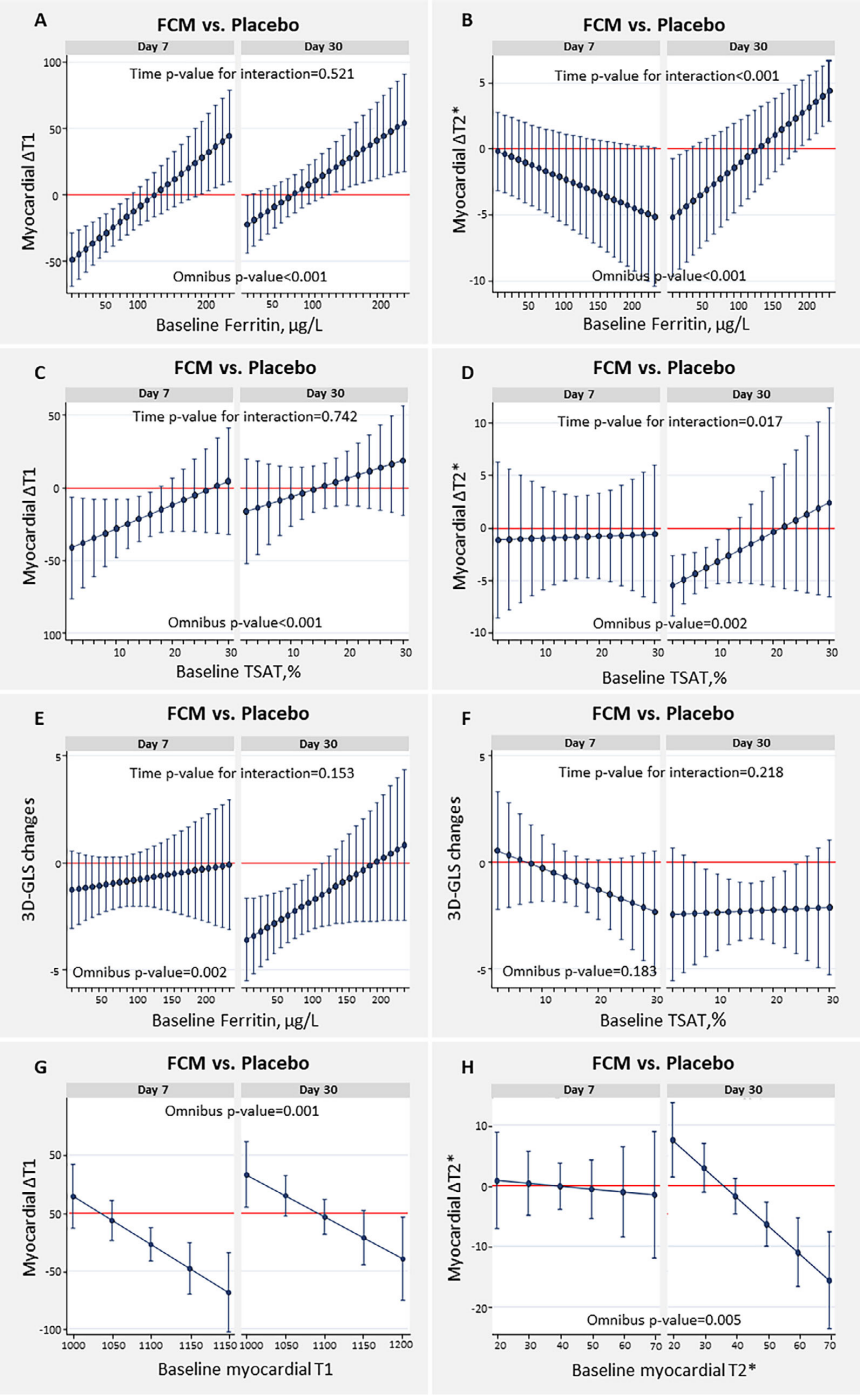
Baseline ferrokinetics and changes in cardiac magnetic resonance parameters following administration of ferric carboxymaltose (FCM) in the Myocardial‐IRON trial. (*A*) Between treatment‐effect (FCM vs. placebo) on change in myocardial T1 (∆T1) across baseline ferritin. (*B*) Between treatment‐effect (FCM vs. placebo) on change in myocardial T2* (∆T2*) across baseline ferritin. (*C*) Between treatment‐effect (FCM vs. placebo) on change in myocardial T1 (∆T1) across baseline transferrin saturation (TSAT). (*D*) Between treatment‐effect (FCM vs. placebo) on change in myocardial T2* (∆T2*) across baseline TSAT. (*E*) Baseline ferritin and changes in three‐dimensional global longitudinal strain (3D‐GLS) following administration of FCM. (*F*) Baseline TSAT and changes in 3D‐GLS following administration of FCM. (*G*) Between treatment‐effect (FCM vs. placebo) on change in myocardial T1 (∆T1) across baseline T1. (*H*) Between treatment‐effect (FCM vs. placebo) on change in myocardial T2* (∆T2*) across baseline T2*.

In terms of predictors of myocardial iron uptake following IV iron therapy, between‐treatment comparisons (FCM vs. placebo) in Myocardial‐IRON showed that lower ferritin values at baseline predicted greater myocardial iron uptake evidenced by a greater decrease in myocardial T1 at 7 and 30 days (*Figure* *1A*) and greater decrease in myocardial T2* at 30 days (*Figure* *1B*). Lower TSAT identified those with greater drop in myocardial T1 at 7 days (*Figure* *1C*), and those with greater drop in myocardial T2* at 30 days (*Figure* *1D*). Cut‐off values for ferritin and TSAT at which myocardial uptake diverged from placebo were <90 μg/L and <18%, respectively, when considering ∆T1 as a measure of myocardial iron uptake, and <40 μg/L and <12%, respectively, when using ∆T2* as a measure of myocardial iron uptake. Lower baseline ferritin was also associated with a greater improvement in 3D‐GLS at 30 days (*Figure* *1E*). TSAT did not predict changes in 3D‐GLS (*Figure* *1F*). Higher baseline myocardial T1 also identified those with greater drop in myocardial T1 at 7 and 30 days (*Figure* *1G*), while higher baseline myocardial T2* identified those with greater drop in myocardial T2* at 30 days (*Figure* *1H*).

**Figure 2:**
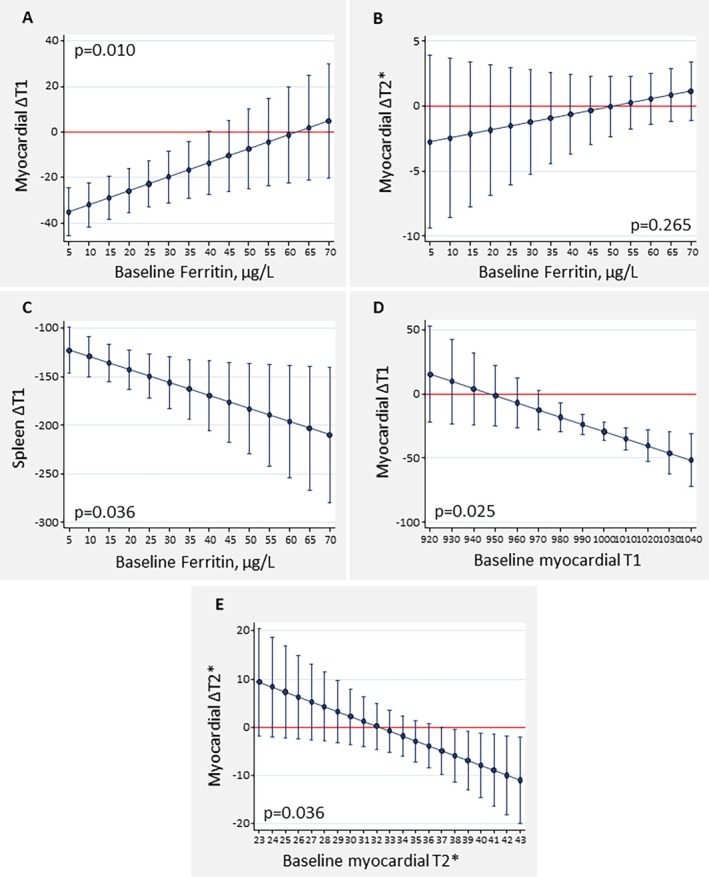
Baseline ferrokinetics and changes in cardiac magnetic resonance parameters following administration of ferric carboxymaltose (FCM) in STUDY. (*A*) Baseline ferritin and changes in myocardial T1 (day 42 vs. baseline) after FCM therapy. (*B*) Baseline ferritin and changes in myocardial T2* (day 42 vs. baseline) after FCM therapy. (*C*) Baseline ferritin and changes in spleen T1 (day 42 vs. baseline) after FCM therapy. (*D*) Baseline myocardial T1 and changes in myocardial T1 mapping (day 42 vs. baseline) after FCM therapy. (*E*) Baseline myocardial T2* and changes in myocardial T2* (day 42 vs. baseline) after FCM therapy.

In STUDY, within‐group comparison showed that lower baseline ferritin identified patients with a greater myocardial iron uptake as evidenced by a greater decrease in myocardial T1 at 42 days (*Figure* *2A*), though this association was not apparent when considering ∆T2* as a measure of myocardial iron uptake (*Figure* *2B*). In contrast, lower baseline ferritin identified those with the least drop in spleen T1 (*Figure* *2C*). Baseline TSAT was not associated with changes in either myocardial T1, T2*, or spleen T1 (data not shown). Higher baseline myocardial T1 and T2* values were significantly associated with greater reductions in T1 and T2* at 42 days, respectively (*Figure* *2D,E*).

## Discussion

**Figure 3:**
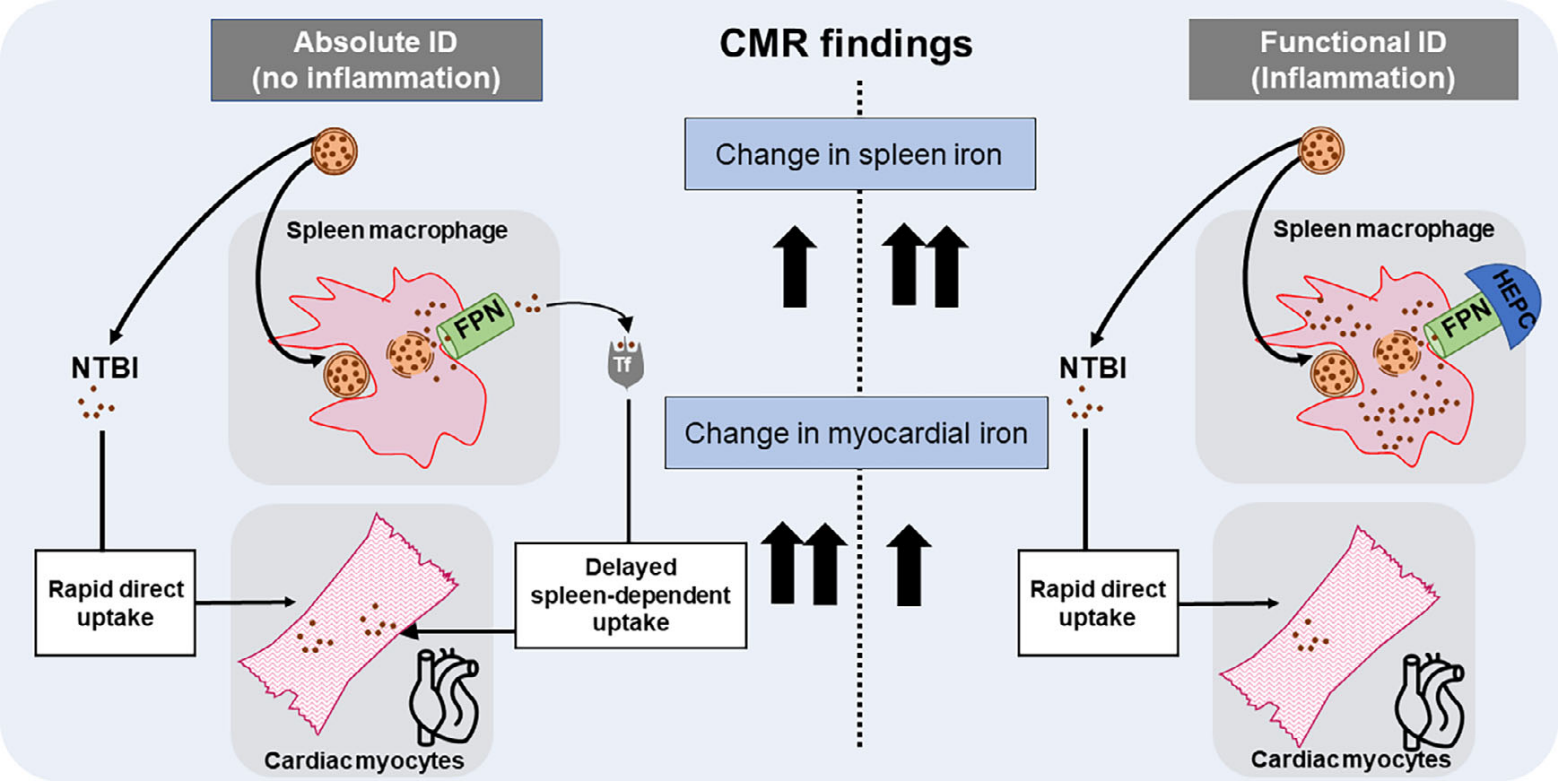
Hypothetical framework for the impact of inflammation on the body's handling of iron from intravenous iron therapy. Iron‐carbohydrate complexes such as ferric carboxymaltose release some iron directly into the circulation as non‐transferrin bound iron (NTBI) which is rapidly and directly taken up by cardiac myocytes, as demonstrated previously by Vera‐Aviles *et al*.^3^ The remainder of iron retained within the carbohydrate complex is taken up by macrophages of the reticuloendothelial system, particularly in the spleen. Here, iron is freed from the carbohydrate complex and exported into the circulation via the iron exporting protein ferroportin (FPN). Upon export into the circulation, iron is loaded onto its chaperone transferrin, and made available for uptake by peripheral tissues including the heart. In the absence of inflammation, low hepcidin levels result in high FPN expression on splenic macrophages, allowing the spleen to redistribute iron from intravenous iron formulations. In the presence of inflammation, high hepcidin (HEPC) levels block FPN on splenic macrophages, causing splenic iron trapping, and limiting the redistribution of iron to peripheral tissues. Thus, in the absence of inflammation, iron from intravenous iron therapies is delivered to the heart both directly (as NTBI) and indirectly via the intermediary of the spleen. However, inflammation blocks the spleen‐dependent pathway of iron delivery, thereby limiting the amount of iron taken up into the myocardium. CMR, cardiac magnetic resonance; ID, iron deficiency.

In patients with or without HF, lower plasma ferritin at baseline predicts greater rise in myocardial iron in the weeks after IV iron therapy with FCM. In terms of underlying mechanisms, spleen data from the STUDY trial point towards a plausible hypothesis. As well as raising plasma ferritin, inflammation also promotes iron retention within spleen macrophages. Indeed, hepcidin, the iron regulatory hormone is induced by inflammation and blocks the iron exporter ferroportin on the surface of splenic macrophages.^4^, ^5^ Our findings that baseline ferritin is a positive predictor of the rise in spleen iron but a negative predictor of the rise in myocardial iron point towards the hypothesis that, in inflamed settings, there is greater trapping of iron within spleen macrophages, which limits iron's subsequent redistribution to peripheral tissues such as the heart (*Figure* *3*). The implication would be that myocardial iron repletion after IV iron therapy would be limited or short‐lived in the setting of functional ID.^6^, ^7^, ^8^, ^9^

There is an ongoing debate on what definition of ID identifies HF patients likely to derive the greatest benefit from IV iron therapy. Recently, responder analyses of IV iron trials suggested that anaemia and functional ID predict greatest benefits.^6^, ^7^, ^8^, ^9^ Our data, on the other hand, suggest that lower ferritin predicts greater myocardial iron repletion, at least in the short term. One interpretation to reconcile these seemingly divergent results is that myocardial iron repletion is not in fact a mechanism for benefit. An alternative interpretation is that IV iron therapy only benefits those with myocardial iron depletion. This idea is supported by our finding that greater myocardial iron uptake from IV iron therapy is seen in those with higher myocardial T1 or T2* at baseline (denoting lower baseline myocardial iron content). Past studies have shown myocardial iron depletion mainly occurs in patients with advanced HF,^10^ but these patients were not largely represented in the aforementioned IV iron trials.

This report generates a plausible hypothesis of how inflammation could modify the impact of IV iron therapy, and highlights the need for a larger study that formally examines the relationship between myocardial iron repletion and improvements in echocardiographic and clinical outcomes across the ferrokinetic spectrum.

The current report has important limitations. First, it is a non‐prespecified analysis and therefore should be considered as a hypothesis‐generating study. Second, it brings together data from two populations that differ from one another in terms of demographics and baseline iron status. Third, limited sample size precludes robust subgroup analysis, for example, according to baseline demographics and medications. Finally, the lack of data from endomyocardial biopsies is another key limitation. Indeed, while the non‐invasive nature of CMR permits within‐patient comparisons of pre‐ versus post‐IV iron therapy, it does not represent a direct quantitation of myocardial iron and may not be fully concordant with it.^11^, ^12^, ^13^

### Funding

J.N. was supported by an unrestricted grant from Vifor Pharma, CIBER Cardiovascular [grant numbers 16/11/00420], Unidad de Investigación Clínica y Ensayos Clínicos INCLIVA Health Research Institute, Spanish Clinical Research Network (SCReN; PT13/0002/0031 and PT17/0017/0003), cofounded by Fondo Europeo de Desarrollo Regional‐Instituto de Salud Carlos III, and Proyectos de Investigación de la Sección de Insuficiencia Cardiaca 2017 from Sociedad Española de Cardiología. M.V.A., S.K., and S.L.L. were funded by a Medical Research Council Senior Research Fellowship awarded to S.L.L. (MR/V009567/1/) and the British Heart Foundation Centre for Research Excellence (HSR00031 and RE/18/3/34214).

**Conflict of interest**: J.N. reports previous research funding from Vifor Pharma, AstraZeneca and honoraria on lectures or advisory boards from Alleviant, AstraZeneca, Boehringer Ingelheim, Bayer, Novartis, Novo Nordisk, Pfizer, Rovi, and Vifor Pharma. S.L.L. reports previous research funding from Vifor Pharma, personal honoraria on a lecture from Pharmacosmos and consultancy fees from Disc Medicine and ScholarRock. All other authors have nothing to disclose.
